# The complete chloroplast genome of *Catalpa* ‘Bairihua’, a hybrid variety with multi season flowering

**DOI:** 10.1080/23802359.2020.1788445

**Published:** 2020-07-11

**Authors:** Wen-Qing Li, Yi-Zeng Lu, Xiao-Man Xie, Yi Han, Ning Wang, Tao Sun, Li-Jiang Liu, Yan Wang, Zhen-Jie Zhuang, Guang-Chen Gao, Tao Liu, Li-Jun Zhao

**Affiliations:** Shandong Provincial Center of Forest Tree Germplasm Resources, Jinan, China

**Keywords:** *Catalpa* ‘Bairihua’, chloroplast genome, phylogeny

## Abstract

The complete chloroplast genome of *Catalpa* ‘Bairihua’ a hybrid variety with multi season flowering obtained from hybrid progeny of *C. bungei ‘*Luoqiu Sihao’ (*C. bungei* ‘01’ × *C. bungei* ‘Changguo Qiu’) and *C. fargesii* f. *duclouxii* was first sequenced with the Illumina HiSeq 2000 platform. Which was 158,210 bp in length with a typical quadruple structure and contained a large single copy (LSC: 84,928 bp) region and a small single copy (SSC: 12,664 bp) region that were separated by a pair of inverted repeats (IRa, b: 30,309 bp) region. The GC content of the whole chloroplast genome is 38.1%. A total of 130 genes was annotated in the complete chloroplast genome, including 85 protein-coding genes, 37 tRNA genes and 8rRNA genes. ML phylogenetic analysis by comparing with 39 chloroplast genomes of the Bignoniaceae indicated that *Catalpa* ‘Bairihua’ was close to *Tecomaria capensis*.

Tree species of *Catalpa* Scop. are frequently used as important timber, ornamental and medicinal plant. *Catalpa* ‘Bairihua’ (variety right No. of China 20180198) is one of the excellent material obtained through the hybrid breeding of *C. bungei* C. A. Mey. *‘*Luoqiu Sihao’ (*C. bungei* C. A. Mey. ‘01’ × *C. bungei* C. A. Mey. ‘Changguo Qiu’) (Science and Technology Development Center of State Forestry Administration, 2014) and *C. fargesii* Bur. f. *duclouxii* (Dode) Gilmour. It can bloom more than 3 times in the year of asexual propagation as crops and flowering lasted for 3 months, while still maintained a medium-high rate of growth. It would be an ideal material for genetic breeding and bioengineering research of *Catalpa* with the establishment of tissue culture (Yi et al. 2017) and genetic transformation system of *Catalpa*. However its genetic information was lack. Up to now, the chloroplast genome of *Catalpa* was still blank too. Through the chloroplast genome mining of *Catalpa* ‘Bairihua’, it was not only helpful to understand the potential value of *Catalpa* ‘Bairihua’ in genetic breeding and Bioengineering, but also helpful to the genetic resources mining of *Catalpa*.

The fresh leaves of *Catalpa* ‘Bairihua’ were collected from the living individual permanently conserved in the *Catalpa* gene bank (36.762°N, 117.454°E) and which leaf tissue could be shared with other researchers. Total genomic DNA (Saved in DNA library of Shandong Provincial Center of Forest Tree Germplasm Resources with the code of lzy2019CBBRH01) was extracted by the Plant DNA extraction Kit (TIANGEN, Beijing, China) according to the requirements of the reagent company.

Paired-end reads were sequenced on the Illumina HiSeq 2000 platform. We used the MITObim 1.8 to assemble the whole chloroplast genome (Hahn et al. 2013). And which was annotated in DOGMA (http://dogma.ccbb.utexas.edu/). A maximum-likelihood (ML) tree with 100 bootstrap replicates was inferred using TreeBeST 1.9.2 (Vilella et al. 2009).The chloroplast genome of *Catalpa* ‘Bairihua’ (GenBank accession number MT591528) is the first whole chloroplast genome of *Catalpa*. It was a typical quadruple structure with 158,210 bp in length and contained a large single copy (LSC: 84,928 bp) region and a small single copy (SSC: 12,664 bp) region, which were separated by a pair of inverted repeats (IRa, b: 30,309 bp) region. The GC content of the whole chloroplast genome was 38.1%. The whole chloroplast genome consists of 130 genes, including 85 protein-coding genes, 37 tRNA genes, and 8 rRNAgenes. Among these, 7 genes had a single intron and *ndh*B, *rpl*2 had double genes. Two genes (*ycf*3and *clp*P) have two introns, and the others have no introns. It has four Trans cutting genes of *rps*12 and five genes (*ycf*1, *rrn*4.5, *rrn*5, *rrn*16, *rrn*23) have two copies in the regions of IRa and IRb. To explore the evolution status of *Catalpa* ‘Bairihua’, the phylogenetic tree was constructed with MAFFT v7.307 (Kazutaka and Standley 2013) based on the other chloroplast genomes of publicly available 38 species of the Bignoniaceae. The phylogenetic analysis revealed that *Catalpa* ‘Bairihua’ is close to the *Tecomaria capensis* (Figure 1).

**Figure 1.**
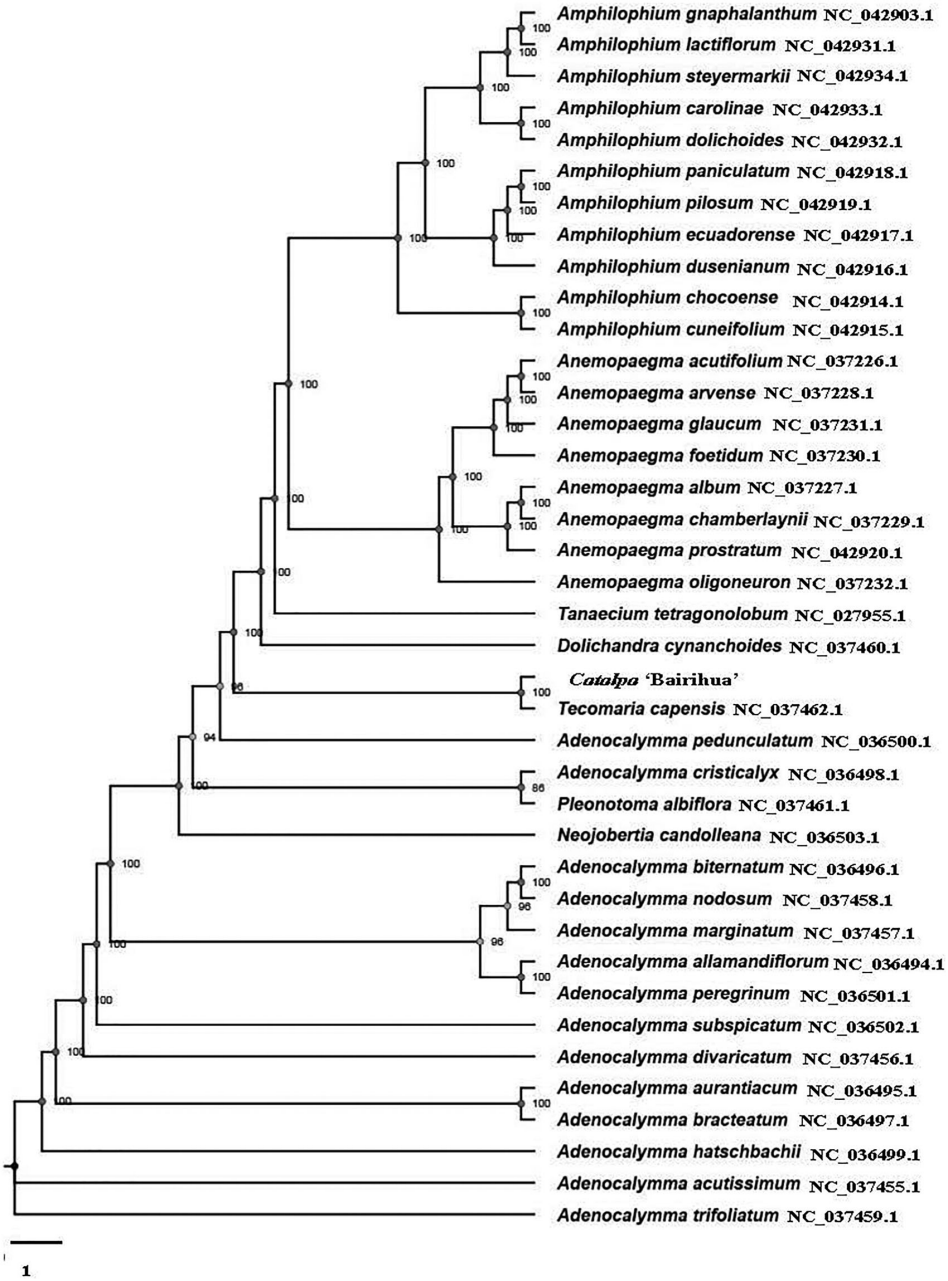
Maximum-likelihood (ML) analysis of *Catalpa* ‘Bairihua’ (*C. bungei ‘*Luoqiu Sihao’ (*C. bungei* ‘01’×*C. bungei* ‘Changguo Qiu’) × *C. fargesii* f. *duclouxii*) and other related species of the Bignoniaceae based on the complete chloroplast genome sequence. The accession numbers were showed in the figure, and the numbers behind each node are bootstrap support values.

## Data Availability

The data that support the findings of this study are available in GenBank of NCBI at https://www.ncbi.nlm.nih.gov/genbank/, GenBank accession number MT591529.
